# Autosomal Dominant Hypocalcemia With Hypercalciuria Due to a Calcium-Sensing Receptor Gain-of-Function Mutation: A Pediatric Case Report

**DOI:** 10.7759/cureus.111214

**Published:** 2026-06-20

**Authors:** Tasneim Makki, Anwar Al-Omairi, Saif Al Yaarubi, Naji Al Dhawi

**Affiliations:** 1 Pediatric Nephrology, Sultan Qaboos University Hospital, Muscat, OMN; 2 Child Health, Sultan Qaboos University Hospital, Muscat, OMN; 3 Pediatrics and Child Health, Oman Medical Specialty Board, Muscat, OMN

**Keywords:** autosomal dominant hypocalcemia, calcium-sensing receptor (casr), casr mutation, congenital hypoparathyroidism, gain-of-function mutation, hypercalciuria, hypocalcemia, nephrocalcinosis (nc), pediatric nephrology

## Abstract

Autosomal dominant hypocalcemia with hypercalciuria (ADHH) is a rare genetic disorder caused by gain-of-function mutations in the calcium-sensing receptor (CaSR) gene. It is characterized by hypocalcemia, inappropriately low parathyroid hormone (PTH), and renal hypercalciuria.

We describe a 13-year-old boy who was diagnosed with congenital hypoparathyroidism, persistent hypercalciuria, and mild medullary nephrocalcinosis. Despite calcium and vitamin D supplementation, hypercalciuria was aggravated. Genetic testing revealed a heterozygous c.2518G>A (p.Gly840Ser) mutation in the CaSR gene, classified as likely pathogenic (American College of Medical Genetics and Genomics (ACMG) Class 4). Supplementation was discontinued, and hydrochlorothiazide and potassium citrate were initiated to reduce urinary calcium and alkalinize urine. At the one-year follow-up, renal function remained preserved with no progression of nephrocalcinosis.

This case underscores the importance of considering ADHH in children with hypocalcemia, suppressed PTH, and renal hypercalciuria. Early genetic testing prevents misdiagnosis and enables targeted therapy to mitigate long-term renal complications.

## Introduction

Autosomal dominant hypocalcemia with hypercalciuria (ADHH), also termed autosomal dominant hypoparathyroidism type 1 (ADH1), is a rare, inherited form of hypoparathyroidism caused by gain-of-function variants in the calcium-sensing receptor (CaSR) gene, which increase receptor sensitivity to extracellular calcium and suppress parathyroid hormone (PTH) secretion at normal calcium levels [1-3]. Patients typically present with hypocalcemia, hyperphosphatemia, inappropriately low or low-normal PTH, and often hypercalciuria due to both low PTH and the activation of renal tubular CaSR [1,2,4]. Clinical severity is highly variable, ranging from asymptomatic hypocalcemia to seizures and end-organ damage such as nephrocalcinosis, nephrolithiasis, reduced kidney function, and intracranial calcifications [1,3,5-8]. Conventional management with oral calcium and active vitamin D can correct symptoms of hypocalcemia but frequently worsens hypercalciuria and renal complications, prompting interest in alternative approaches including thiazide diuretics, PTH analogs, and calcilytic CaSR antagonists that target the underlying pathophysiology [1,6,7,9-11].

Herein, we report a pediatric case of ADHH caused by the heterozygous c.2518G>A (p.Gly840Ser) CaSR variant, a mutation with limited prior documentation in the literature, who was initially misdiagnosed as idiopathic hypoparathyroidism and managed with calcium and vitamin D supplementation. This case is distinctive in that targeted genetic diagnosis prompted a timely and complete therapeutic reversal, with subsequent stabilization of renal disease on hydrochlorothiazide and potassium citrate, illustrating how molecular confirmation can fundamentally redirect clinical management and prevent irreversible renal injury in children.

## Case presentation

**Figure 1:**
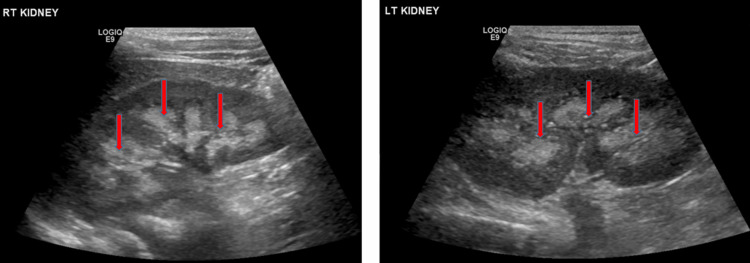
Renal ultrasound demonstrating mild bilateral medullary nephrocalcinosis

A 13-year-old boy was referred to pediatric nephrology for persistent hypercalciuria and bilateral medullary nephrocalcinosis. He was diagnosed with congenital hypoparathyroidism at three months of age based on recurrent hypocalcemia (initial serum calcium: 1.6 mmol/L) and suppressed PTH levels and was managed with oral calcium carbonate and alfacalcidol. There was no known family history of hypocalcemia, renal stones, or nephrocalcinosis. Throughout childhood, he remained clinically well without tetany, seizures, muscle cramps, or other neurological symptoms; Chvostek and Trousseau signs were consistently negative on examination. Despite escalating therapy, serum calcium levels remained at the low end of normal, while urinary calcium excretion progressively increased. At eight years of age, the urine calcium-to-creatinine ratio peaked at 1.9 mmol/mmol (normal for age: <0.2 mmol/mmol). Serum phosphate was consistently mildly elevated (2.2-2.5 mmol/L), and magnesium was within the normal range. Serum creatinine remained normal throughout, with estimated glomerular filtration rate (eGFR) estimated using the Schwartz formula consistently exceeding 90 mL/min/1.73 m², monitored every six months. Renal ultrasound demonstrated mild bilateral medullary nephrocalcinosis predominantly involving the renal medullary pyramids (Figure 1).

Given the paradoxical combination of hypocalcemia, suppressed PTH, and progressive hypercalciuria despite escalating calcium and vitamin D therapy, a CaSR-related disorder was suspected. Whole-exome sequencing (CentoXome®, CENTOGENE, Rostock, Germany) identified a heterozygous c.2518G>A (p.Gly840Ser) mutation in the distal transmembrane domain (TM7) of the CaSR gene, at the boundary with the intracellular C-terminal tail, previously reported in association with ADHH and classified as likely pathogenic based on the American College of Medical Genetics and Genomics (ACMG) criteria (Class 4) [1,5]. Following genetic confirmation, calcium and vitamin D analogs were discontinued. Hydrochlorothiazide had been trialed empirically prior to diagnosis but was discontinued without sustained benefit, likely reflecting suboptimal dosing in the absence of a confirmed diagnosis and a targeted regimen. It was subsequently re-initiated at a weight-appropriate dose alongside potassium citrate. This combination aimed to reduce urinary calcium excretion and alkalinize the urine to improve calcium solubility. At the 12-month follow-up, renal imaging remained stable with no progression of nephrocalcinosis, and the patient remained asymptomatic with preserved renal function. Serum calcium and PTH levels remained relatively stable without clinically significant worsening, serum phosphate remained within an acceptable range, and urinary calcium excretion improved compared with baseline, as shown in Table 1.

**Table 1:** Key biochemical and urinary investigations during the clinical course PTH: parathyroid hormone; Ca: calcium; Cr: creatinine

Timeline	Total calcium: 2.20-2.70 (mmol/L)	Phosphate: 1.10-1.95 (mmol/L)	PTH: 1.6-6.9 (pmol/L)	Urine Ca/Cr: <0.20 (mmol/mmol)	Clinical significance
Month 0	1.85	1.93	0.2	0.32	Initial suppressed PTH
Age 3 years 8 months (Year 3, Month 8)	1.93	2.01	0.5	1.9	Persistent hypocalcemia with low PTH
Age 11 years (Year 11)	2.05	2.11	-	1.6	Marked hypercalciuria
Age 12 years 1 month (Year 12, Month 1)	2.06	2.20	-	1.92	Progressive hypercalciuria
Age 12 years 7 months (Year 12, Month 7)	2.01	2.49	-	0.9	Genetic diagnosis confirmed
Age 13 years 10 months (Year 13, Month 10)	2.05	2.44	-	0.6	Improvement after targeted therapy
Age 14 years 4 months (Year 14, Month 4)	2.08	2.36	0.8	-	Stable follow-up

## Discussion

ADHH, also known as ADH1, is a rare inherited disorder caused by gain-of-function variants in CaSR that increase CaSR sensitivity to extracellular calcium, leading to hypocalcemia, inappropriately low or normal PTH, and relative or absolute hypercalciuria [1-3]. These abnormalities reflect the key role of CaSR in PTH regulation and renal calcium handling [2,4]. Misdiagnosis as idiopathic hypoparathyroidism is well recognized and may result in conventional treatment with calcium and active vitamin D, which frequently aggravates hypercalciuria and increases the risk of nephrocalcinosis, nephrolithiasis, and renal impairment [1,7,11]. This paradoxical worsening occurs because activated renal tubular CaSR directly suppresses calcium reabsorption in the thick ascending limb and distal nephron, independent of PTH; exogenous calcium and active vitamin D further increase the filtered calcium load without correcting the underlying tubular defect, thereby amplifying urinary calcium losses and accelerating nephrocalcinosis [4,7].

In this case, the patient's asymptomatic hypocalcemia, low PTH, and hypercalciuria are typical for ADH1/ADHH [1,3]. The heterozygous CaSR c.2518G>A (p.Gly840Ser) variant lies within the distal transmembrane domain, a recognized hotspot for activating ADH1 mutations that enhance CaSR signaling [2,5]. Systematic ADH1 reviews report many such transmembrane missense variants, often without strict genotype-phenotype correlation but consistently associated with hypocalcemia and hypercalciuria [1,3].

Differentiating ADHH from other hypocalcemic disorders is critical because standard hypoparathyroidism therapy can worsen renal outcomes [1,7]. Contemporary ADH1 recommendations favor conservative targets (maintaining calcium at the low-normal or slightly subnormal range to avoid symptoms) and minimizing calcium/active vitamin D doses to limit hypercalciuria and renal calcifications [4,7]. Thiazide diuretics have been shown to significantly reduce urinary calcium excretion and help maintain acceptable serum calcium in patients with CaSR gain-of-function mutations by promoting sodium-calcium co-reabsorption in the distal convoluted tubule [4,6]. Potassium citrate was added as a complementary measure: citrate inhibits calcium crystal nucleation and growth in the tubular lumen, urinary alkalinization increases calcium solubility, and potassium citrate offsets the hypokalemia and mild metabolic acidosis that thiazide diuretics can induce, making this a particularly rational combination [9].

Timely genetic testing is emphasized in recent prevalence, management, and pathophysiology studies as essential to establish an accurate diagnosis, distinguish ADHH from other forms of hypoparathyroidism, guide individualized therapy, and enable family counseling [1,7].

The absence of in vitro functional testing for this specific p.Gly840Ser variant is a limitation; however, its location within a well-established activating region, the patient's characteristic biochemical profile, and prior reports of similar transmembrane gain-of-function variants strongly support likely pathogenicity [1,2,5]. Longitudinal data from ADHH cohorts show that renal complications (nephrocalcinosis, nephrolithiasis, and chronic kidney disease) are common, particularly in hypercalciuric patients and after conventional therapy, underscoring the need for ongoing follow-up with regular urinary calcium measurements and periodic renal imaging [1,3,9,11].

In conclusion, ADHH due to CaSR gain-of-function mutations should be considered in children with hypocalcemia, low or normal PTH, and hypercalciuria [1,3]. Genetic confirmation is central to selecting appropriate, kidney-sparing management strategies and may substantially influence long-term renal outcomes [1,7].

## Conclusions

This case highlights the importance of recognizing ADHH in children presenting with hypocalcemia, suppressed PTH, and renal hypercalciuria. Genetic testing of the CaSR gene is critical to avoid misdiagnosis and prevent inappropriate supplementation, which may worsen renal outcomes. Timely diagnosis and targeted therapy, including thiazide diuretics and potassium citrate, can reduce the risk of long-term renal complications and improve patient outcomes.
